# Draft Genome Sequence of *Pseudomonas* sp. Strain FEN, Isolated from the Fe- and Organic Matter-Rich Schlöppnerbrunnen Fen

**DOI:** 10.1128/MRA.01017-20

**Published:** 2021-01-14

**Authors:** Rebecca E. Cooper, Carl-Eric Wegner, Kirsten Küsel

**Affiliations:** a Aquatic Geomicrobiology, Institute of Biodiversity, Friedrich Schiller University Jena, Jena, Germany; b German Centre for Integrative Biodiversity Research Halle-Jena-Leipzig, Leipzig, Germany; University of Arizona

## Abstract

Here, we report the draft genome sequence of *Pseudomonas* sp. strain FEN, a nonfluorescent siderophore producer that was isolated from the Schlöppnerbrunnen fen, which is characterized by high concentrations of Fe, dissolved organic matter (DOM), and Fe-DOM complexes. This draft genome sequence provides insight into the mechanisms of siderophore biosynthesis and siderophore-mediated iron uptake by this bacterium.

## ANNOUNCEMENT

*Pseudomonas* spp. are Gram-negative bacteria belonging to the *Pseudomonadaceae* family within the class *Gammaproteobacteria* and are characterized by metabolic versatility and colonization of a wide range of diverse habitats (1–3). A single nonfluorescent, siderophore-producing *Pseudomonas* sp. strain was isolated from soil samples collected from the moderately acidic (pH 4.5 to 6.0), Fe-rich minerotrophic Schlöppnerbrunnen fen located in the Fichtelgebirge (northern Bavaria, Germany; 50°7′54″N, 11°52′51″E). Enrichments were grown by inoculating Fe gradient tubes, prepared as described previously (4, 5), with 25 μl soil slurry. Briefly, the gradient tubes contained an FeS bottom layer (1:1 FeS mixed with modified Wolfe’s mineral medium [MWMM] [ATCC medium 2672] and 3% [wt/vol] agarose) overlaid with 0.2% (wt/vol) agarose-stabilized MWMM supplemented with Wolfe’s vitamin solution, 1 mM ferulic acid, and 1 mM syringic acid. Active enrichment cultures were transferred to new gradient tubes every 4 to 6 weeks, and the dilution-to-extinction method was used to obtain the *Pseudomonas* sp. strain FEN isolate.

*Pseudomonas* sp. FEN stock cultures were cultivated at room temperature on solid agar plates containing PS medium (ATCC medium 3). Overnight shaking cultures grown in 100 ml PS medium at room temperature were inoculated from a single colony picked from the PS plate. Overnight cultures were used for genomic DNA extraction with the GenElute bacterial genomic DNA kit (Sigma-Aldrich, Taufkirchen, Germany). Whole-genome sequencing was performed on the RS II platform (Pacific Biosciences [PacBio], Menlo Park, CA) according to the standard manufacturer's protocol. Briefly, a 10- to 20-kb library was prepared and sequenced on a PacBio RS II sequencer using C4-P6 chemistry on single-molecule real-time (SMRT) cells, with a 180-min collection protocol. Sequencing and subsequent filtering with Hierarchical Genome Assembly Process 4 (HGAP4) resulted in 153,019 reads with an average read length of 5,607 bp. The maximum read length was 74,202 bp. The genome was assembled *de novo* with HGAP4 using default parameters except for the estimated genome size (6.0 Mbp) (6). Genome sequence annotation and gene identification were performed with RASTtk v2.0 using default parameters (7–9).

**Fig 1:**
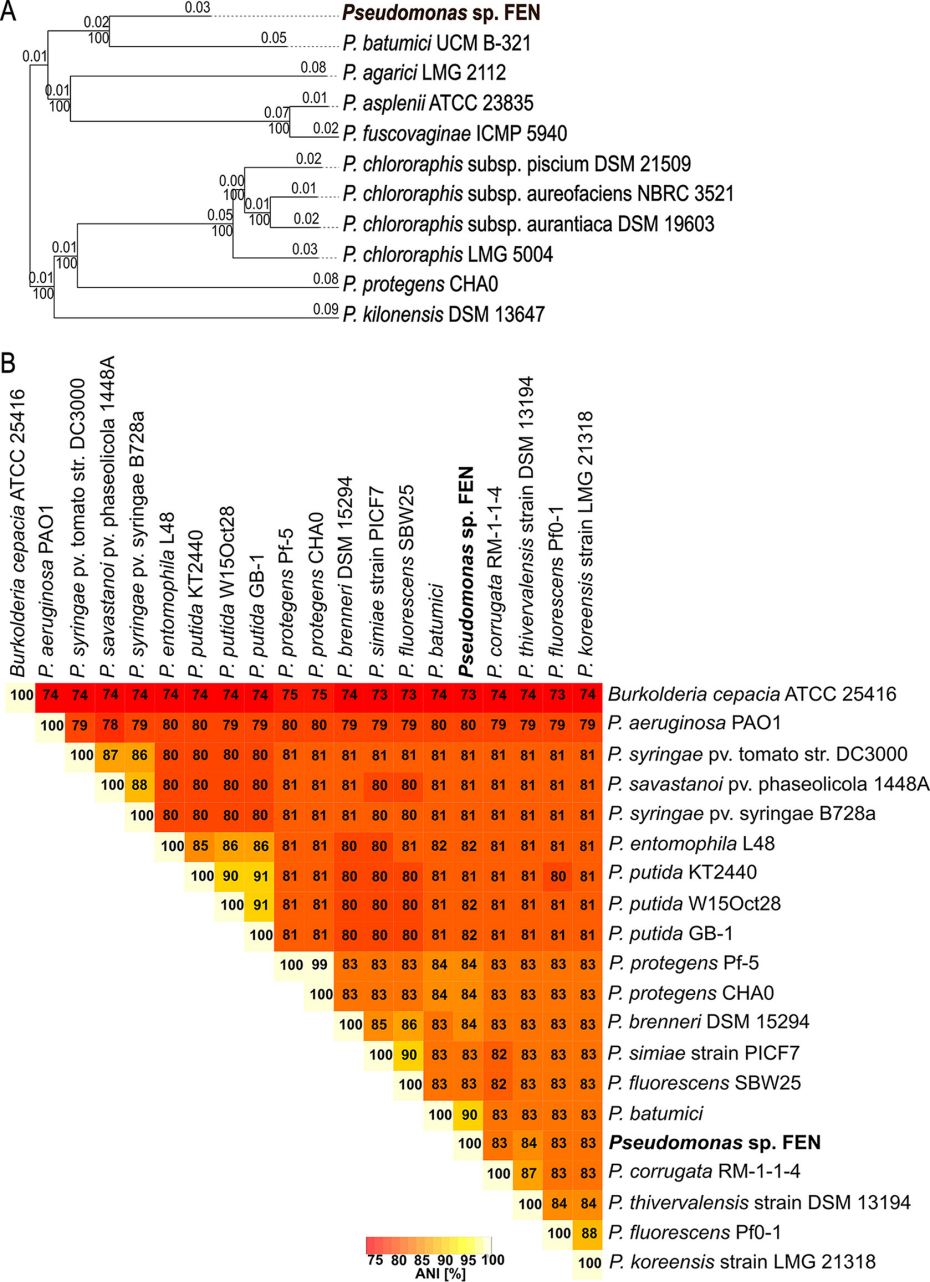
Phylogenetic analysis based on pairwise comparisons of *Pseudomonas* sp. FEN versus type strain genomes and ANI-based analysis of publicly available genome sequences of siderophore-producing *Pseudomonas* sp. strains. (A) Phylogenetic tree based on whole-genome comparisons using the TYGS. The phylogenetic tree was inferred using FASTME v2.1.6.1 from genome-based distance phylogeny (GBDP) distances calculated from closely related genome sequences. The branch lengths are scaled based on GBDP distances. The numbers above each branch represent GBDP pseudo-bootstrap values of >60% from 100 replications. The tree was rooted at the midpoint. (B) ANI genome-based distance matrix calculator output, based on pairwise ANI between different siderophore-producing *Pseudomonas* sp. strains within the phylum *Proteobacteria* with publicly available genomes, including the *Pseudomonas* sp. FEN isolate described here. All ANI values are shown in the matrix. Pseudomonas cepacia was renamed Burkholderia cepacia ATCC 25416 and was used in the ANI-based analysis due to its siderophore-producing phenotype.

The draft genome of *Pseudomonas* sp. FEN was assembled into 36 contigs totaling 6.79 Mbp, with a G+C content of 61.1%, an *N*_50_ value of 576,490 bp, and an *L*_50_ value of 4. Mapping of filtered raw reads onto the assembly revealed 90-fold coverage. The draft genome contains 6,294 coding sequences and 98 non-protein-coding genes. Initial cloning and 16S rRNA gene-based sequencing approaches indicated that the isolate was most closely related to Pseudomonas fluorescens. However, recent advances in genomics and taxonomic classifications, specifically implementation of average nucleotide identity (ANI)-based comparisons, standardized taxonomic classifications (Genome Taxonomy Database [GTDB] taxonomy) (10, 11), and the automated genome-based taxonomic analysis tool Type Strain Genome Server (TYGS) (12), revealed that the isolate is more closely related to the proposed type strain Pseudomonas batumici UCM B-321 (3, 13), rather than to any of the publicly available P. fluorescens genome sequences (Fig. 1). According to GTDB-Tk v0.3.2 analysis (10, 11, 14–19), the ANI between P. batumici and *Pseudomonas* sp. FEN is 90.56%, based on an aligned fraction of 0.73 (proportion of regions displaying significant similarity). This taxonomic classification clarifies why fluorescent siderophores, such as pyoverdine (20), were not detected (21).

Analysis of the *Pseudomonas* sp. FEN genome revealed putative genes encoding pathways for siderophore biosynthesis, Fe-transport systems, Fe-siderophore sensor proteins, siderophore receptor proteins, and ferrichrome (hydroxamate siderophore) uptake systems and receptors. Several genes encoding nonspecific siderophore uptake systems were identified, suggesting that these systems play a role in the uptake of exogenous siderophores, including pyoverdines. Homologs of the ferric Fe-ABC transport system (*pitADC*) and a ferrous Fe-transport system (*efuUOB*) were also identified. The draft genome of *Pseudomonas* sp. FEN provides supporting evidence that this microorganism is capable of siderophore production, import, and export, as well as detection of exogenous siderophores. Further investigations may reveal additional genes or regulatory mechanisms involved in siderophore production and uptake in environments characterized by high concentrations of dissolved organic matter (DOM) and Fe-DOM complexes, such as the Schlöppnerbrunnen fen.

### Data availability.

The sequencing reads and assemblies for this whole-genome shotgun project are available in the European Nucleotide Archive (ENA) repository under the BioProject accession number PRJEB40039. The version described in this paper is the first version. The BioSample number is SAMEA7280114, and the individual genome assembly is available under the accession number CAJFDC010000000.
